# ATM Mutations Benefit Bladder Cancer Patients Treated With Immune Checkpoint Inhibitors by Acting on the Tumor Immune Microenvironment

**DOI:** 10.3389/fgene.2020.00933

**Published:** 2020-08-14

**Authors:** Ruibin Yi, Anqi Lin, Manming Cao, Abai Xu, Peng Luo, Jian Zhang

**Affiliations:** ^1^Department of Oncology, Zhujiang Hospital, Southern Medical University, Guangzhou, China; ^2^Department of Urology, Zhujiang Hospital, Southern Medical University, Guangzhou, China

**Keywords:** ATM, bladder cancer, immune checkpoint inhibitors, DDR, tumor microenvironment

## Abstract

Immune checkpoint inhibitors (ICIs) have shown promising results in bladder cancer (BC). However, only some patients respond to ICIs. DNA repair defects (DDR) play an important role in the therapeutic response of bladder cancer. Therefore, we aimed to elucidate the association between ICIs in bladder cancer and ataxia telangiectasia mutated (ATM), a core component of the DNA repair system. From a collected immunotherapy cohort (*n* = 210) and The Cancer Genome Atlas (TCGA)-Bladder cancer cohort, which were both retrieved from publicly available resources, we performed a series of analyses to evaluate the prognostic value and potential mechanism of ATM in bladder cancer immunotherapy. We found that ATM-mutant (ATM-MT) bladder cancer patients derived greater benefit from ICIs [overall survival (OS), hazard ratio (HR) = 0.28, [95% confidence interval (CI), 0.16 to 0.51], *P* = 0.007] and showed a higher mutation load (*P* < 0.05) and immunogenicity (*P* < 0.05) than ATM-wild-type (ATM-WT) patients. The immune inflammatory response to antigenic stimulation, the regulation of the IFN pathway and the macrophage activation pathway were significantly enriched in the ATM-MT group (NES > 1, *P* < 0.05), while insulin-like growth factor receptor signaling pathways and vasculogenesis were significantly downregulated (NES < −1, *P* < 0.05). ATM mutation significantly upregulated the number of DNA damage repair pathway gene mutations (*P* < 0.05). ATM mutations resulted in increased bladder cancer sensitivity to 29 drugs (*P* < 0.05), including cisplatin and BMS-536924, an IGF-1R inhibitor. Our results demonstrate the importance of ATM as a prognostic signature in bladder cancer and reveal that ATM may impact the effects of ICIs by acting on the tumor immune microenvironment.

## Introduction

As one of the most common cancers in the world, bladder cancer (BC) is more common in men than in women (Antoni et al., 2017). Approximately 80–90% of bladder cancers are urothelial carcinomas (UCs), and the remainder include adenocarcinomas, squamous cell carcinomas and other rare histologies (Willis and Kamat, 2015). Currently, the first-line treatment for patients with locally progressive or metastatic UC is cisplatin-based chemotherapy (von der Maase et al., 2000). However, almost half of patients still show recurrence or progression (Oing et al., 2016). Notably, the high mutational load of bladder cancer confers high immunogenicity, which makes bladder cancer a good candidate for immunotherapy strategies, such as immune checkpoint blockade (ICB) (Chabanon et al., 2016). In 2016, immune checkpoint inhibitors (ICIs) were approved for the treatment of bladder cancer patients with advanced refractory disease or who were ineligible platinum chemotherapy, improving patient prognosis (Zibelman et al., 2016). Although ICB therapy against the checkpoint axis of programmed death ligand-1 (pd-l1)/pd-1 has shown some promising results as a UC therapy (Sharma et al., 2017), only 20–30% of UC patients have a partial or complete response to ICI therapy (Mariathasan et al., 2018). Thus, the identification of new potential biomarkers to guide targeted clinical therapy and thus increase the proportion of patients with bladder cancer responsive to immunotherapy is urgently needed.

DNA repair gene defects play an important role in bladder cancer occurrence, development, therapeutic response and prognosis. DNA repair gene somatic mutations are often found in bladder cancer (Cancer Genome Atlas Research Network, 2014) and are associated with a relatively good response to cisplatin-class neoadjuvant chemotherapy (Plimack et al., 2015). Liu et al. demonstrated that changes in the ERCC2 gene may predict the response of bladder cancer to cisplatin neoadjuvant chemotherapy (Freedman and Pfeiffer, 2016). Teo et al. showed that DNA damage response (DDR) gene mutations are associated with a relatively good prognosis in platinum-treated advanced bladder cancer (Teo et al., 2017). These studies confirm the strong link between DDR mutations and bladder cancer. In addition, the deficiency in the DNA repair machinery has recently been reported to be associated with increased response to ICI (Le et al., 2015, 2017). Teo et al. (2018) found that DDR gene mutation was associated with an increased ICI response rate. These results suggest that the DDR pathway may play an important role in ICI therapy.

Ataxia telangiectasia mutated (ATM) is a core component of the DNA repair system that activates the ATM-enhanced homologous recombination repair pathway upon recognition of a DNA double-strand break (Weber and Ryan, 2015). Recently, inhibiting the DDR pathway has become a common treatment strategy in many cancers (Cremona and Behrens, 2014), and the ATM signaling pathway is known to play an important part in the development of breast cancer, germ cell carcinoma and other cancers (Shiloh and Kastan, 2001; Jin and Oh, 2019). Plimack et al. (2015) demonstrated that changes in the ATM, RB1, and FANCC genes can predict the efficacy of neocisplatin chemotherapy in bladder cancer. Additional studies have reported that ATM gene defects are one of the mechanisms of spontaneous tumor-infiltrating lymphocyte (TIL) infiltration in breast and ovarian cancer (Rooney et al., 2015). In addition, other studies have suggested that DDR-deficient cancer cells exhibit IFN response activation and cell recruitment via the chemokines CCL5 and CXCL10 (Hartlova et al., 2015; Nakad and Schumacher, 2016). These studies may explain some of the possible mechanisms underlying the utility of DNA repair gene mutations in predicting cancer prognosis, but the significance of DNA repair gene mutations as biomarkers for predicting treatment efficacy in bladder cancer has not been well defined. At present, there is still insufficient research available to be able to stratify patients according to treatments and evaluate the effects of specific DDR gene mutations on predicting the efficacy of immunotherapy in bladder cancer.

Based on this information, we evaluated the relationship between ATM gene mutations and the prognosis of patients receiving ICIs. According to the mutation status of the ATM gene, patients who received ICI treatment (anti-pd-1/pd-l1 therapies) and had mutation data available were divided into ATM-mutant (MT) and ATM-wild-type (WT) groups. We found that the ATM-MT bladder cancer patients had better benefits from ICI therapy and showed a higher mutation load and immunogenicity. In addition, we explored the differences in pathway activation between the ATM-MT bladder cancer patients and ATM-WT patients through functional enrichment analysis to explain the effect of ATM on the efficacy of ICIs and the tumor microenvironment (TME).

## Materials and Methods

### Relationship Between ATM Gene Mutation and Immunotherapy Prognosis in Bladder Cancer

We collected an immunotherapy cohort with clinical and mutational data (reported by Samstein et al., 2019), within which 210 bladder cancer patients with mutational data received ICI therapy (anti-pd-1/pd-l1 therapies). To evaluate the relationship between ATM gene mutations and the prognosis of patients receiving ICIs, we divided the patients into ATM-MT and ATM-WT groups according to their ATM mutational status and performed Kaplan–Meier (KM) analysis. In addition, we also collected The Cancer Genome Atlas (TCGA)-Bladder cancer cohort with somatic mutation and survival data from the Genomic Data Commons^1^ using the R package TCGAbiolinks (Colaprico et al., 2016) for download. Then, we used cBioportal (Cerami et al., 2012) to download the survival data [OS, disease-free survival (DFS), and progression-free survival (PFS)] of the TCGA-Bladder cancer cohort and conducted similar KM analysis according to the ATM mutational status.

### Mutation Characteristics and Tumor Immunogenicity

To identify the gene mutation characteristics and tumor immunogenicity of ATM-MT bladder cancer patients, we analyzed the immunotherapy cohort (Samstein et al., 2019) somatic mutation data generated by targeted next-generation sequencing (NGS; MSK-IMPACT). The neoantigen mutational load (neoantigen loading; NAL) data from the TCGA-Bladder cancer cohort was also evaluated (Thorsson et al., 2018). Next, we downloaded whole-exome sequencing (WES) data for bladder cancer cell lines from Genomics of Drug Sensitivity in Cancer (GDSC) (Yang et al., 2013). To quantify the tumor mutational burdens (TMBs) of the immunotherapy cohort (Samstein et al., 2019), TCGA-Bladder cancer cohort and GDSC-Bladder cancer cell line cohort, we referred to other literature (Chalmers et al., 2017) and treated the non-synonymous mutations in each cohort as raw mutations and divided by 38 Mb. Finally, the R package ComplexHeatmap (Gu et al., 2016) was used to visualize the mutation rates in the top 20 genes and the clinical features of the immunotherapy cohort and TCGA-Bladder cancer cohort. In addition, the R package Maftools (Mayakonda et al., 2018) was used to visualize the correlation between ATM and the mutation rates in the top 20 genes of the TCGA-Bladder cancer cohort. The ATM gene mutation sites in the immunotherapy cohort and TCGA-Bladder cancer cohort were visualized using the R package Maftools.

### Analysis of Immune Characteristics and Drug Sensitivity

To further compare the relationship between ATM gene mutation status and the TME, we compared the mRNA expression levels of immune-related genes in the ATM-MT and ATM-WT patients of TCGA-Bladder cancer. Immune-related genes and their functional classifications and immune-related scores have been reported (Hao et al., 2018). We also compared the mRNA expression levels of immune cell-related genes in the ATM-MT and ATM-WT patients of TCGA-Bladder cancer (Thorsson et al., 2018). The expression levels of all these genes were quantified as fragments per kilobase of exon model per million mapped fragments (FPKM) and log2-transformed. The cutoff value was set to logFC = 1. In addition, TCGAbiolinks was used to download the TCGA-Bladder cancer gene expression data (Illumina HiSeq, RNASeq), and CIBERSORT^2^ (Newman et al., 2015) and xCell^3^ (Aran et al., 2017) were used to compare the immune cell infiltration proportion in the ATM-WT and ATM-MT patients of TCGA-Bladder cancer. The R package software MCP counter (Becht et al., 2016) was used to quantify the absolute abundance of immune and stromal cells. Finally, we downloaded the drug-sensitivity data for the bladder cancer cell lines from GDSC and analyzed the differences in sensitivity to different drugs according to the ATM gene mutation status.

### Copy Number Alteration (CNV) Analysis

To analyze Copy number alteration (CNV) mutations in the ATM gene in the TCGA-Bladder cancer patients, we used Broad GDAC Firehose^4^ to download Affymetrix 6.0 SNP microarray data (hg19; germline/potential false-positive calls were removed). The downloaded CNV segments were subjected to GISTIC2.0 analysis by GenePattern^5^ (Reich et al., 2006). Except for a couple parameters (e.g., confidence level of 0.99; X chromosomes not excluded before analysis), the GISTIC2.0 analysis used default settings. Finally, the results of the GISTIC2.0 analysis were visualized using the R package Maftools (Mayakonda et al., 2018).

### Pathway Enrichment Analysis and DDR Pathway Mutation Number Analysis

First, we used the R package edgeR to conduct differential analysis of the gene expression data (raw count) of TCGA-Bladder cancer downloaded with TCGAbiolink (Robinson et al., 2010). The ClusterProfiler R package (Yu et al., 2012) was used to perform gene annotation enrichment analysis, and a gene set enrichment analysis (GSEA) dataset was obtained from the MSigDB database of Broad Institute (Subramanian et al., 2005). *P* < 0.05 was considered to be significant for gene ontology (GO) terms, Kyoto Encyclopedia of Genes and Genomes (KEGG) pathways and Reactome pathways. In addition, we evaluated and compared the difference in non-synonymous mutation numbers in the DDR pathways between the ATM-MT and ATM-WT groups in the immunotherapy cohort, TCGA-Bladder cancer cohort, and GDSC-Bladder cancer cell line dataset. The gene set of DDR pathways was derived from the DDR pathway gene set of the MSigDB database of the Broad Institute (Subramanian et al., 2005) (see Additional File: Table S1).

### Statistical Analysis

We used the Mann–Whitney U test to compare the differences in TMB, NAL, immune cell abundance, immune-related gene expression, age, microsatellite instability (MSI) score and number of mutations in DDR pathway components between ATM-MT and ATM-WT groups. In addition, we used Fisher’s exact test to determine the relationships between ATM gene mutation status and mutation status of the top 20 genes, tumor sample type (primary/metastatic), sex, and treatment (single drug/combination) in the immunotherapy cohort. We also used Fisher’s exact test to determine the relationships between ATM mutations and sex, race, ethnicity and clinical stage in the TCGA-Bladder cancer cohort. Survival analysis was performed with the KM method and log-rank test. P < 0.05 was considered statistically significant, and all statistical tests were bilateral. All statistical tests and visualized analyses were performed with R software (version 3.6.1). In addition, the R package ggpubr was used for visualizing boxplots (Kassambara, 2018). The false discovery rate (FDR) for CNV visualization was 0.05.

## Results

### Association Between the ATM Status and Clinical Outcomes

**FIGURE 1:**
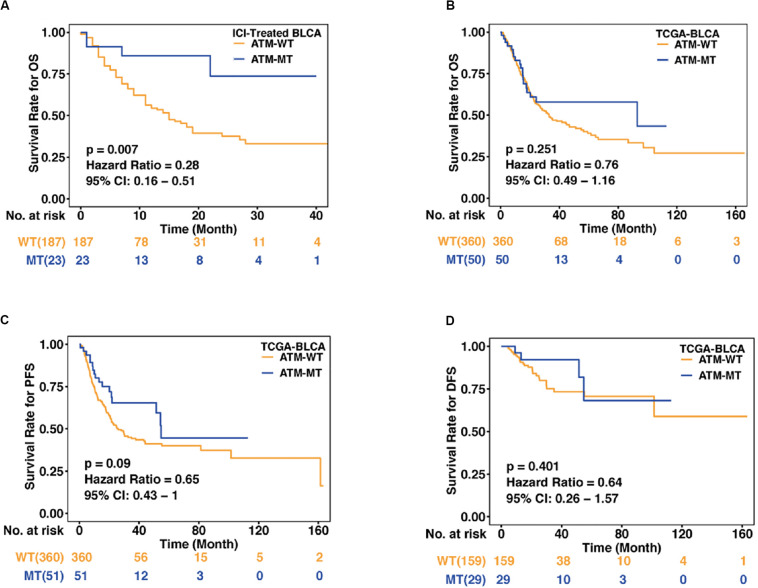
Association between the ATM status and clinical outcomes in bladder cancer. **(A)** Kaplan–Meier curves comparing the overall survival (OS) of patients with ATM-MT with that of patients with ATM-WT in the immunotherapy cohort [*n* = 210, hazard ratio (HR) = 0.28, [95% confidence interval (CI), 0.16 to 0.51], *P* = 0.007]. ATM-MT, ATM mutant type; ATM-WT, ATM wild type; BLCA, bladder cancer. **(B)** Kaplan–Meier curves comparing the OS of patients with ATM-MT with that of patients with ATM-WT in the TCGA-BLCA cohort (*n* = 410, HR = 0.76, [95% CI, 0.49 to 1.16], *P* = 0.251). **(C)** Kaplan–Meier curves comparing the progression-free survival (PFS) of patients with ATM-MT with that of patients with ATM-WT in the TCGA-BLCA cohort (*n* = 411, HR = 0.65, [95% CI, 0.43 to 1.00], *P* = 0.09). **(D)** Kaplan–Meier curves comparing the disease-free survival (DFS) of patients with ATM-MT with that of patients with ATM-WT in the TCGA-BLCA cohort (*n* = 188, HR = 0.64, [95% CI, 0.26 to 1.57], *P* = 0.401).

To evaluate the relationship between ATM gene mutations and the prognosis of bladder cancer patients who received ICI treatment, we collected an immunotherapy cohort with clinical data and mutational data (Samstein et al., 2019). The baseline characteristics of the immunotherapy cohort are summarized in Table 1. According to their ATM gene mutation status, bladder cancer patients with mutation data treated with ICIs (anti-pd-1/pd-l1 therapies) (*n* = 210) were divided into ATM-MT and ATM-WT groups, and then KM analysis was performed. We found that the OS time of the ATM-MT bladder cancer patients treated with ICIs was longer than that of the ATM-WT patients [Figure 1A, hazard ratio (HR) = 0.28, [95% confidence interval (CI), 0.16 to 0.51], *P* = 0.007]. In addition, we downloaded the TCGA-Bladder cancer cohort somatic mutation and survival data from the Genomic Data Commons (see footnote 1) and similarly performed KM analysis according to the ATM mutation status. We found that ATM gene mutation did not predict prognosis in the TCGA cohort patients who did not receive ICI treatment (Figure 1B: OS, *n* = 410, HR = 0.76 [95% CI, 0.49 to 1.16], *P* = 0.251; Figure 1C: PFS, *n* = 411, HR = 0.65 [95% CI, 0.43 to 1.00], *P* = 0.09; Figure 1D: DFS, *n* = 188, HR = 0.64 [95% CI, 0.26 to 1.57], *P* = 0.401). Therefore, among bladder cancer patients treated with ICIs, patients with ATM gene mutation exhibited a better prognosis than those with ATM-WT.

**TABLE 1:** The baseline characteristics of the immunotherapy cohort.

**Characteristics**	**N (%)**
**Gender**	
Male	159 (76)
Female	51 (24)
**Age decade**	
31–50	12 (6)
50–60	43 (21)
61–70	66 (31)
>70	89 (42)
**Sample Type**	
Primary	121 (58)
Metastasis	89 (42)
**Drug class**	
PD-1/PD-L1	187 (89)
Combo	23 (11)
**Survival events**	
Survival	92 (44)
Died	118 (56)
**ATM Status**	
Mutated type	187 (89)
Wild type	23 (11)
Tumor Mutational Burden (Median)	8.8
MSI Score (Median)	0.3

### Relationships Between the ATM Mutation Status and Clinical Phenotype and Mutation Load in Bladder Cancer

**FIGURE 2:**
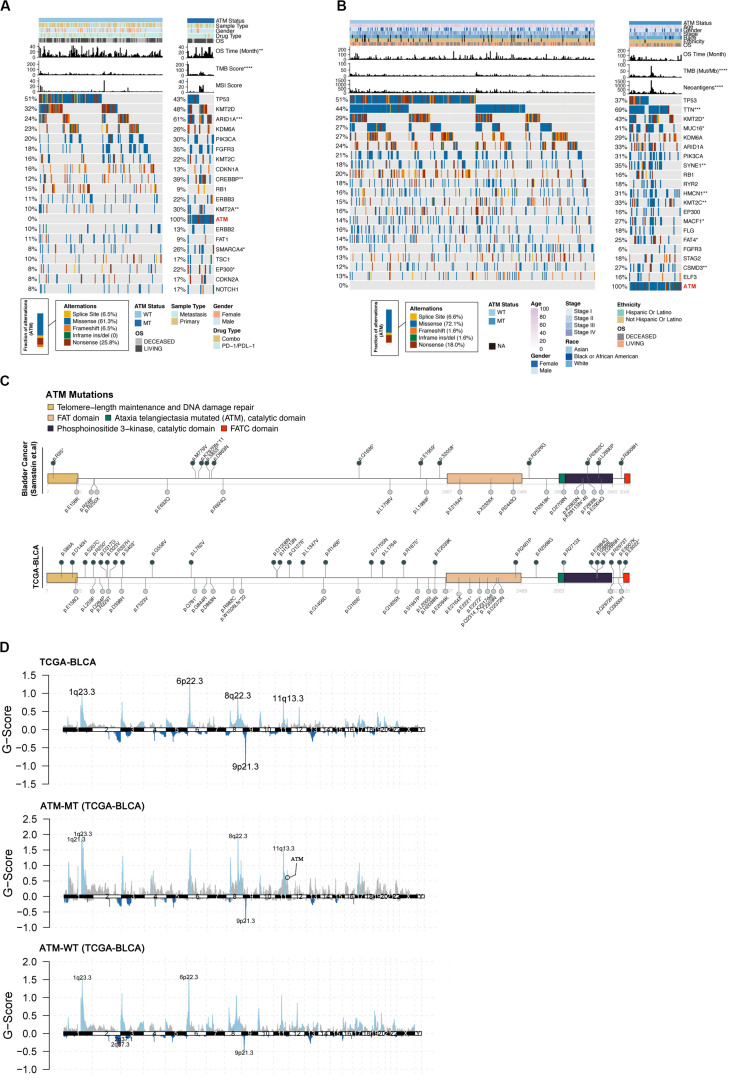
Relationship between the ATM status and clinical phenotype. **(A)** Top 20 significantly mutated genes in the immunotherapy cohort of bladder cancer patients. Samples were ordered based on the somatic non-synonymous mutation burden, and genes were ranked by mutation frequencies (left panel). The ATM status, sample type (metastasis/primary), sex, drug type, OS, OS time (months), TMB score and MSI score are annotated in order in the top panel. **(B)** Top 20 significantly mutated genes in the TCGA-BLCA cohort. ATM status, age, sex, stage, ethnicity, OS, OS time (months), TMB and neoantigen load are annotated in order in the top panel. **(C)** The somatic mutations in ATM were evenly distributed in both the immunotherapy cohort and the TCGA-BLCA cohort. **(D)** Maftools was used to visualize the copy number alteration (CNV) analysis based on GISTIC2.0 of the TCGA-BLCA cohort.

To further explore the relationships between the ATM mutation status and clinical phenotype and mutation load in bladder cancer, we compared the differences in the mutation status in the top 20 genes, tumor sample type (primary/metastatic), sex, race, ethnicity, treatment (single drug/combination) and clinical stage between the ATM-MT and ATM-WT patients in the immunotherapy cohort and TCGA-Bladder cancer cohort. The results showed that the ATM-MT group exhibited a better prognosis and a higher TMB score and that the mutation frequencies of ARID1A, CREBBP, KMT2A, SMARCA4, and EP300T were significantly higher in the ATM-MT group than in the ATM-WT group in the immunotherapy cohort (Figure 2A, *P* < 0.05). In the TCGA-Bladder cancer cohort, there was no significant difference in prognosis between the ATM-MT group and the ATM-WT group. The ATM-MT group had a higher neoantigen load, and the mutation frequencies of TTN, KMT20, MUC16, SYNE1, HMCN1, KMT2C, MACF1, FAT4, and CSMD3 were significantly higher in the ATM-MT group than in the ATM-WT group (Figure 2B, *P* < 0.05). Correlation analysis showed that ATM had co-occurrence with TTN, KMT2C, and CSMD3 genes (*P* < 0.05), and tended to be mutually exclusive with FGFR3 (*P* < 0.1) (Supplementary Figure S1). Additionally, we analyzed the ATM mutation sites and CNV. The results showed that the ATM mutation sites were evenly distributed among all five important regions (Figure 2C). Both ATM-MT group and ATM-WT group have a large copy number variation regardless of the status of ATM. While comparing to the ATM-WT group, we found that there were more CNVs in the ATM-MT group (Figure 2D). The significantly amplified regions in ATM-MT patients included 1q21.3, 1q23.3, 8q22.3, and 11q13.3, of which 11q13.3 span the CCND1 (cyclin D1) gene. A significant deletion in ATM-MT patients included 9p21.3, spanning the tumor suppressor genes CDKN2A and CDKN2B (Figure 2D).

### ATM Mutation Enhances Immunogenicity and Activates Antitumor Immunity

**FIGURE 3:**
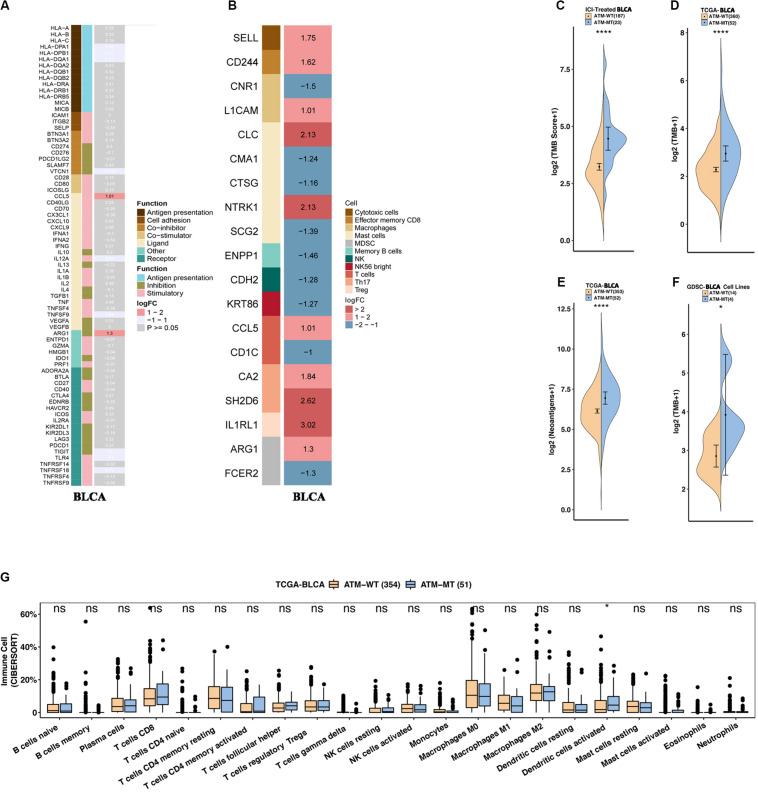
ATM mutation was associated with enhanced tumor immunogenicity and activated antitumor immunity. **(A)** Heatmap showing average changes in the expression levels of immune-related gene between the ATM-MT and ATM-WT patients in the TCGA-BLCA cohort. The genes corresponding to the same lymphocyte or function are identified by the same color on the left side of the squares, and each square with an exact number represents the logFC of a gene, filled with different back colors, i.e., from red to gray. The logFC values marked in black font indicate that the absolute value of logFC is ≥1 with statistical significance (*P* < 0.05), while the logFC values in white font are non-significant (*P* > 0.05). Using Mann–Whitney U test tested the differences in tumor-infiltrating lymphocytes and immune-related gene expression between ATM-MT and ATM-WT samples. **(B)** Heatmap showing average changes in the expression levels of immune-cell-related gene between the ATM-MT and ATM-WT patients in the TCGA-BLCA cohort. The genes corresponding to the different cell types are identified by the different colors on the left side of the squares, and each square with an exact number represents the logFC of a gene, filled with different back colors, i.e., from red to blue. The logFC values marked in black font indicate that the absolute value of logFC is ≥1 with statistical significance (*P* < 0.05). Using Mann–Whitney U test tested the differences in immune-cell-related gene expression between ATM-MT and ATM-WT samples. **(C)** Comparison of the tumor mutational burden between the ATM-MT and ATM-WT tumors in the immunotherapy cohort. **(D)** Comparison of the tumor mutational burden between the ATM-MT and ATM-WT tumors in the TCGA-BLCA cohort. **(E)** Comparison of the neoantigen load between the ATM-MT and ATM-WT tumors in the TCGA-BLCA cohort. **(F)** Comparison of the tumor mutational burden between the ATM-MT and ATM-WT tumors in the GDSC-Bladder cancer cell line dataset. **(G)** CIBERSORT analyses quantifying the proportion of 22 immune cells in the ATM-MT and ATM-WT tumors in the TCGA-BLCA cohort. **(C–G)**, *p < 0.05; ****p < 0.0001.

To determine how ATM mutations enhance the immunogenicity of bladder cancer and activate antitumor immunity, we compared the expression differences in 74 immune-related genes (antigen presentation/stimulation/inhibition) and immune cell-related genes between the ATM-MT group and AMT-WT group. The results showed that in the ATM-MT group, the expression of the immune-related genes CCL5 and AGE1 was significantly increased (logFC > 1, adjusted *P* < 0.05). Among the immune cell-related genes, the expression of mast cell-related CLC and NTRK1, T helper (Th) 17 cell-related SH2D6, and regulatory T (Treg) cell-related IL1RL1 was significantly upregulated (logFC > 1, adjusted *P* < 0.05) (Figures 3A,B). Moreover, the ATM-MT group showed a higher mutation load and stronger antigen-specific immunity than the ATM-WT group in the immunotherapy cohort, TCGA-Bladder cancer cohort and GDSC-Bladder cancer cell line dataset (Figures 3C–F, *P* < 0.05). Next, we used CIBERSORT to evaluate the data and then compared the levels of 22 immune cells between the ATM-MT and ATM-WT groups. The results showed that the proportion of activated dendritic cells was significantly upregulated in the ATM-MT group (Figure 3G, *P* < 0.05). In addition, xCell analyses showed that the proportion of CD4 + Tem cells was upregulated in the ATM-MT group (Supplementary Figure S2A, *P* < 0.05), while there were no significance in MCP counter analyses (Supplementary Figure S2B).

### Functional Enrichment Pathways Associated With ATM Mutations

**FIGURE 4:**
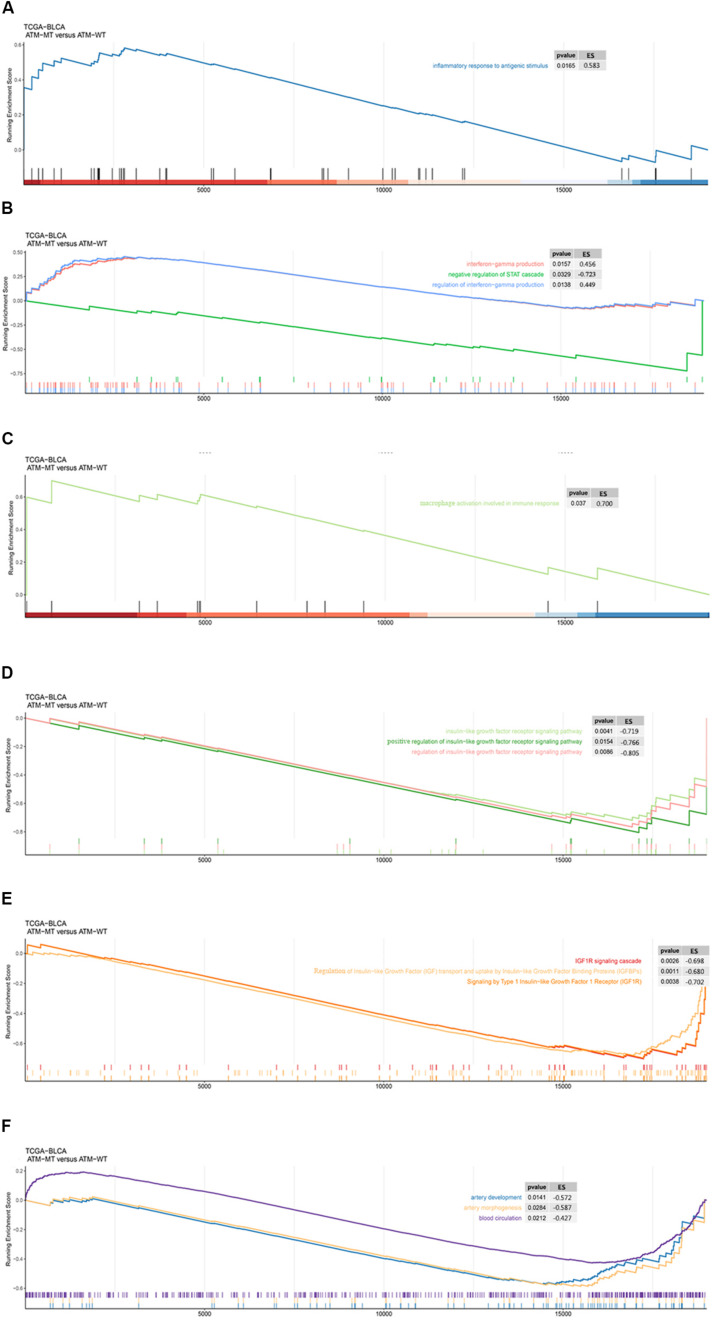
GSEA of the functional enrichment pathways of the ATM-MT and ATM-WT groups in the TCGA-BLCA cohort and GDSC-Bladder cancer cell line dataset. **(A)** The inflammatory response in the antigen pathway was significantly enriched in the ATM-MT group. **(B)** Interferon-gamma production and regulation pathways were significantly enriched in the ATM-MT group. **(C)** Macrophage activation involved in the immune response pathway was significantly enriched in the ATM-MT group. **(D)** The insulin-like growth factor receptor signaling pathway was significantly downregulated in the ATM-MT group. **(E)** The insulin-like growth factor receptor signaling pathway was significantly downregulated in the ATM-MT group. **(F)** Angiogenesis-related pathways were significantly downregulated in the ATM-MT group.

**FIGURE 5:**
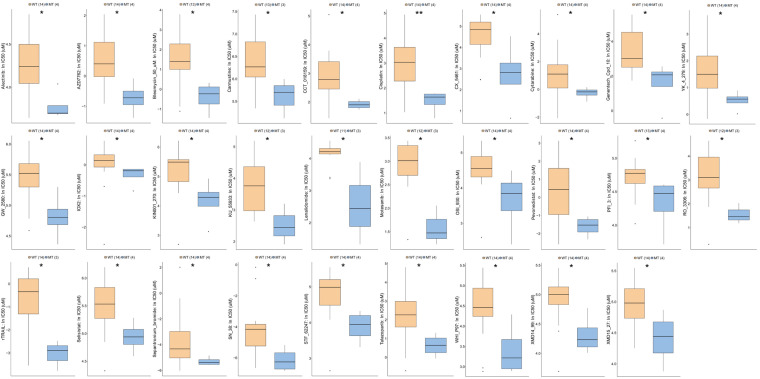
Comparison of drug sensitivity between ATM-MT and ATM-WT in the GDSC-Bladder cancer cell line cohort. We observed that the sensitivity to 29 drugs were increased in the ATM-MT group (*P* < 0.05), including Alectinib, AZD7762, Bleomycin, Carmustine, CCT_018159, Cisplatin, CX_5461, Cytarabine, Genentech_Cpd_10, YK_4_279, GW_2580, IOX2, KIN001_270, KU_55933, Lenalidomide, Motesanib, OSI_930, Pevonedistat, PFI_3, RO_3306, rTRAIL, Selisistat, Sepantronium_bromide, SN_38, STF_62247, Talazoparlb, WHI_P97, XMD14_99, and XMD15_27. IC50s reported in GDSC were log-transformed. Using the Mann–Whitney U test tested the differences in the ln(IC50) values of different drugs between the ATM-MT and ATM-WT cell lines. *P < 0.05; **P < 0.01.

Next, we analyzed the functional enrichment pathways of the ATM-MT and ATM-WT groups in the TCGA-Bladder cancer cohort and GDSC dataset by GSEA. The results showed that the immune inflammatory response to antigenic stimulation, the regulation of the IFN pathway and the macrophage activation pathway were significantly enriched in the ATM-MT group (NES > 1, *P* < 0.05) (Figures 4A–C). Insulin-like growth factor receptor signaling pathways and vasculogenesis were significantly downregulated in the ATM-MT group (NES < −1, *P* < 0.05) (Figures 4D–F). Additionally, we analyzed the effect of ATM mutation on the sensitivity of bladder cancer cells to drug therapy in the GDSC cohort. We found that ATM mutation resulted in increased bladder cancer sensitivity to 29 drugs, including cisplatin (*P* < 0.05), BMS-536924, an IGF-1R inhibitor (*P* < 0.05), motesanib, a VEGFR inhibitor (*P* < 0.05), and WHI-P97, a JAK inhibitor (*P* < 0.05) (Figure 5). In combination with the significant downregulation of insulin-like growth factor receptor expression in the ATM-MT group shown in the GSEA analysis described above, BMS-536924 may have a synergistic effect with ATM mutation in inhibiting IGF-1R.

### ATM Mutation and Mutated DDR Pathway Gene Number Variation

**FIGURE 6:**
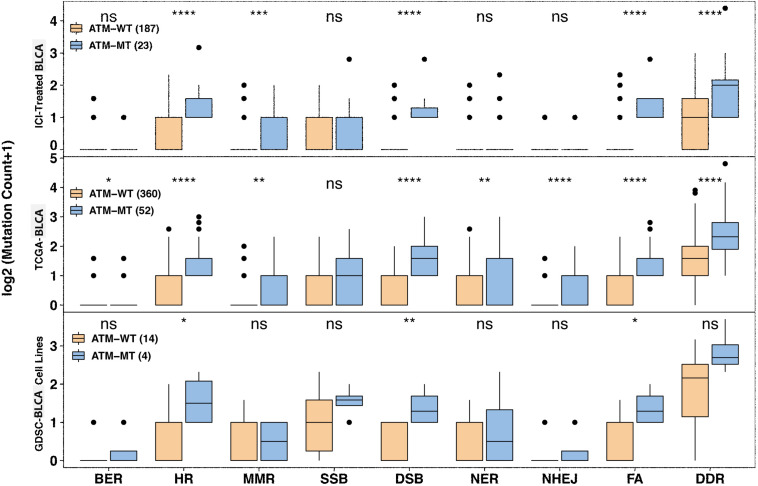
Association between ATM mutation and mutated DNA damage repair pathway gene number variation. Mutated DNA damage repair pathway gene number variation increased in the ATM-MT group in the immunotherapy, TCGA-BLCA and GDSC-Bladder cancer cell line cohorts. The *x* axis indicates eight DDR-related pathways. BER, base excision repair; HR, homologous recombination; MMR, mismatch repair; SSB, single-stranded DNA binding; DSB, double-strand break repair; NER, nucleotide excision repair; NHEJ, non-homologous end-joining; FA, Fanconi anemia. *P < 0.05; **P < 0.01; ***P < 0.001; ****P < 0.0001.

Since DNA repair gene defects play an important role in the occurrence, development, therapeutic response and prognosis of bladder cancer, we further explored the role of ATM mutations in DDR pathways. The eight gene sets related to DNA repair gene defects (DDR) came from MSigDB (Supplementary Table S1). We analyzed the relationship between ATM mutations and gene mutations in the eight DDR-related pathways in the immunotherapy cohort, TCGA-Bladder cancer cohort and GDSC-Bladder cancer cell line dataset. We found that patients with ATM alterations in homologous recombination (HR) (*P* < 0.0001), MMR (*P* < 0.001), DSB (*P* < 0.0001), and FA (*P* < 0.0001) showed comparably high mutation counts than ATM intact patients in the immunotherapy cohort. However, SSB failed to show the significant difference in all cohorts (Figure 6).

## Discussion

Recently, the high mutational load of bladder cancer confers high immunogenicity, which makes bladder cancer a good candidate for immunotherapy strategies. Fan et al. (2019) conducted a meta-analysis of 14 clinical trials and showed that PD-1/PD-L1 treatment was effective with acceptable incidence of treatment-related adverse events. Unfortunately, no effective biomarker has been found to predict the efficacy of immunotherapy in bladder cancer patients. Therefore, we collected an immunotherapy cohort and the TCGA-Bladder cancer cohort containing clinical data and mutation data. Through survival analysis, we found that the patients in the ATM-MT group in the immunotherapy cohort had a significantly better immunotherapy prognosis than those in the ATM-WT group. There was no significant difference in the TCGA-Bladder cancer cohort, which may be related to the lack of sufficient information to stratify patients according to treatment. These results suggest that anti-pd-1/pd-l1 ICI therapy is more effective in ATM-MT bladder cancer patients. Through further analysis, we found that ATM is likely to impact the efficacy of ICIs by affecting the tumor immune microenvironment.

We first explored the role of ATM mutations in bladder cancer by evaluating the associations between the gene mutation status and clinical phenotypes in the immunotherapy cohort and TCGA-Bladder cancer cohort. We found that the ATM-MT group showed a higher mutation load and immunogenicity in both cohorts. Compared to the ATM-WT group, ARID1A and TTN mutations were significantly upregulated in the ATM-MT group in the immunotherapy cohort and the TCGA-Bladder cancer cohort, respectively. The different results between two cohorts may be related to the lack of sufficient treatment information to stratify patients in the TCGA-Bladder cancer cohort. Zhu et al. showed that ARID1A and TTN genes were among the 11 genes with frequent mutations in bladder cancer (Zhu et al., 2020), indicating their importance in predicting immune response in bladder cancer, and suggesting that co-mutations of ARID1A or TTN with ATM may be potential molecular biomarkers for predicting the efficacy of immunotherapy for bladder cancer. We further compared the expression differences in immune-related genes (antigen presentation/stimulation/inhibition) and immune cell-related genes between the ATM-MT and ATM-WT groups. The results showed that the expression of the chemokines CCL5 and AGE1 was significantly upregulated in the ATM-MT group. The chemokine CCL5 plays important roles in the chemotaxis and activation of various immune cells, such as monocytes/macrophages, mast cells and T lymphocytes (Lapteva and Huang, 2010; Marques et al., 2013), and has potential clinical application value as an adjuvant to enhance antitumor immunity (Yang et al., 2017; Dugas et al., 2019). Additionally, we found that cytotoxic cells, memory CD8 cells, mast cells, Th17 cells, Treg cells and other immune cell marker molecules were significantly upregulated. Baharlou R et al. showed that Th17 cells might play an important roles in the antitumor immune response in early stage bladder cancer (Baharlou et al., 2015). Next, we used CIBERSORT to further analyze the proportion of 22 immune cell subtypes in the ATM-MT group and found that the proportion of activated dendritic cells was significantly upregulated in the ATM-MT group. Hartana et al. noted that large numbers of T cells and other cytotoxic cells infiltrated a tumor and that the tumor stage of patients was relatively low, suggesting the possibility of using cytotoxic cells as a new immunotherapy regimen. Additionally, bladder cancer cells may reduce the infiltration of CD8 T cells by inhibiting the ICAM-1/TGFβ2 pathway (Hartana et al., 2018a, b). Several studies have shown that various methods of activating the immune activity of dendritic cells in bladder cancer can induce the dendritic cells to initiate an antitumor immune response (Xiu et al., 2018; Domingos-Pereira et al., 2019). Above all, ATM-MT bladder cancer cells are likely to promote the infiltration of cytotoxic cells and Th17 cells into tumor tissues by secreting relatively large amounts of chemokines, such as CCL5, to activate dendritic cells and promote the therapeutic effect of immune checkpoint inhibition.

**FIGURE 7:**
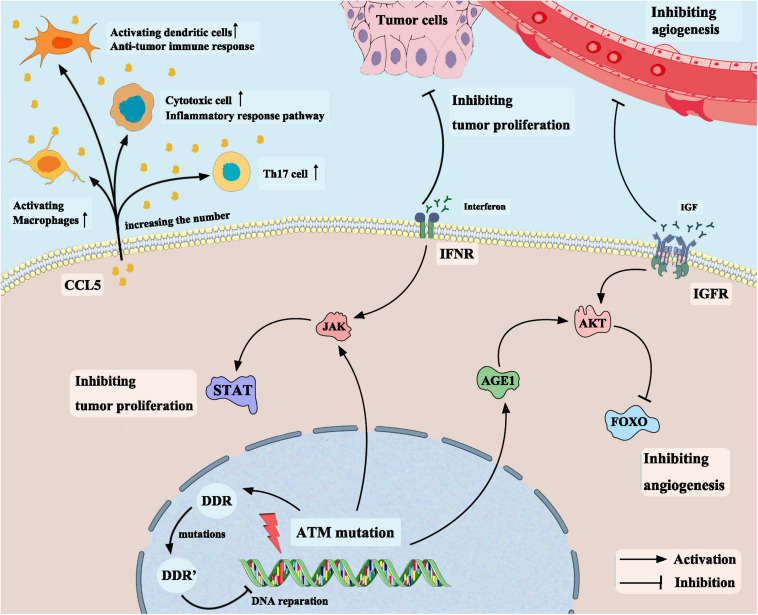
Mechanism of ATM mutation enhancing the efficacy of immune checkpoint inhibitors in bladder cancer based on a hypothetical model.

In addition to the chemokine CCL5, another significantly upregulated gene in the ATM-MT group identified by differential analysis of immune cell-related gene expression was AGE1. The AGE1 gene is a core component of the insulin signaling pathway. Age-1 inactivates the FOXO transcription factor daf-16 by activating downstream AKT, thereby blocking the insulin/insulin-like growth factor receptor signaling pathway (Paradis and Ruvkun, 1998; Kenyon, 2010). Next, we conducted functional pathway enrichment analysis of the TCGA-Bladder cancer cohort by GSEA. The results showed that the insulin-like growth factor receptor pathway was significantly downregulated in the ATM-MT group. Previous studies have demonstrated that high expression of insulin-like growth factor receptor significantly promotes bladder cancer invasion and metastasis through pathways such as the AKT and mitogen-activated protein kinase (MAPK) pathways (Metalli et al., 2010; Safarinejad et al., 2011) and plays an important role in lung cancer, breast cancer and other tumor proliferation (Selinski et al., 2012; Szarvas et al., 2012). Therefore, ATM-MT bladder cancer cells are likely to downregulate the activity of the insulin-like growth factor receptor pathway by upregulating AGE1 gene expression, thereby inhibiting the invasion and metastasis of bladder cancer and improving survival. To confirm our hypothesis, we further analyzed the effect of ATM mutations on bladder cancer by evaluating drug sensitivity data from the GDSC database. The sensitivity to twenty-nine drugs, including cisplatin, BMS-536924, an IGF-1R inhibitor, motesanib, a VEGFR inhibitor, and WHI-P97, a JAK inhibitor, was found to be increased in the ATM-MT group. IGF-1R was reported to be capable of transmitting mitogenic signals to the neoplastic cells and may be a possible target of immunotherapy in the head and neck carcinomas (Friedrich et al., 2010). Martina et al. showed that tamoxifen combined with an IGF-1R inhibitors produced a enhanced response in HER2 positive breast cancer (McDermott et al., 2017). BMS-536924 is an ATP-competitive IGF-1R inhibitor, and the sensitivity to it was enhanced in the ATM-MT group, suggesting that ATM mutation has a synergistic effect with the IGF-1R inhibitor. We inferred that ATM mutation is likely to improve the prognosis of bladder cancer patients by downregulating the activity of the insulin-like growth factor receptor pathway and ATM-MT bladder cancer patients may benefit from the combined application of ICIs and IGF-1R inhibitors, which opens a new approach for clinical treatment (Figure 7). In addition, the sensitivity to VEGFR inhibitor and JAK inhibitor were enhanced in the ATM-MT group, which consistent with the results of GSEA, samely suggesting the synergistic effect of ATM mutation with VEGFR inhibitor and JAK inhibitor. However, our attempt to explain the mechanism of ATM mutation promoting immunotherapy for bladder cancer was based on a hypothetical model, which need to be further verified (Figure 7).

Additionally, in the GSEA results, we also found that the immune inflammatory response to antigens and macrophage activation pathways were significantly enriched in the ATM-MT group, which verified that ATM mutation can promote the antitumor immune response. The IFN signaling pathway was also significantly enriched in the ATM-MT group. A number of studies have demonstrated the antiproliferative effect of IFNγ on bladder cancer cells, and inhibition of IFNγ leads to increased proliferation and invasion (Li et al., 2002; Riemensberger et al., 2002). In addition, the significant downregulation of vasculogenesis pathways in the ATM-MT group suggested that ATM mutations may affect blood perfusion of bladder cancer tumor cells. All these results indicate that ATM mutations affect the immune microenvironment and thus affect the efficacy of ICIs. Moreover, as a core component of the DNA repair system, ATM plays an important role in DNA repair. We found that patients with ATM alteration in DDR-related pathways showed comparably high mutation counts than ATM intact patients, suggesting that ATM mutation patients may show a higher immunogenicity and better efficacy of ICI therapy.

However, there are some limitations to our research. First, although we included two independent cohort studies with clinical data and mutational data, the immunotherapy cohort was relatively small in size, and the analysis results may have statistical errors. Second, while we verified that ATM-MT bladder cancer cells are likely to downregulate the activity of the insulin-like growth factor receptor pathway by upregulating AGE1 gene expression, the functional pathway analysis of ATM mutations needs to be further verified *in vitro* and *in vivo*. Third, we propose that ATM-MT bladder cancer patients may benefit from the combined application of ICIs and IGF-1R inhibitors, which opens a new approach for clinical treatment, but further experimental confirmation is still needed. Finally, this study focused on the prognostic value rather than the predictive value of ATM mutations in patients with bladder cancer who received ICI therapy.

## Conclusion

In conclusion, we found that ATM-MT bladder cancer patients showed greater benefit from ICI therapy than ATM-WT patients in the immunotherapy cohort and TCGA-Bladder cancer cohort. We further found that the ATM-MT group showed a higher mutation load and immunogenicity in both cohorts. By comparing the ATM-MT group immune-related gene and immune cell-related gene expression with that of the ATM-WT group, we found that ATM-MT bladder cancer cells might promote the infiltration of cytotoxic cells and Th17 cells into tumor tissues by secreting increased amounts of the chemokine CCL5 and activating dendritic cells to enhance ICI therapy. Additionally, we found that ATM-MT bladder cancer cells were likely to downregulate the activity of the insulin-like growth factor receptor pathway by upregulating AGE1 gene expression, thus promoting the efficacy of ICIs. Through functional enrichment analysis, we also found that the immune inflammatory response to antigens, the regulation of the IFN signaling pathway and macrophage activation pathways were significantly enriched in ATM-MT bladder cancer patients, while vasculogenesis and insulin-like growth factor receptor signaling pathways were significantly downregulated. We also demonstrated that ATM mutation patients showed comparably high mutation counts than ATM intact patients in DDR-related pathways, thus improving the efficacy of ICI therapy. Finally, by analyzing the GDSC database, we found that ATM mutations resulted in increased bladder cancer sensitivity to 29 drugs, including cisplatin, the first-line treatment for bladder cancer, and BMS-536924, an IGF-1R inhibitor.

## Data Availability Statement

We collected an immunotherapy cohort with clinical and mutational data (reported by Samstein et al., 2019). In addition, we also collected The Cancer Genome Atlas (TCGA)-Bladder cancer cohort with somatic mutation and survival data from the Genomic Data Commons (https://portal.gdc.cancer.gov/). Then, we used cBioportal to download the survival data [overall survival (OS), disease-free survival (DFS), and progression-free survival (PFS)] of the TCGA-Bladder cancer cohort.

## Author Contributions

RY analyzed the data and wrote the manuscript. AL was involved in performing computational coding. MC, AX, PL, and JZ collected the data and designed the study. JZ and PL were involved in the manuscript editing and the supervision of the entire work. All authors have approved the final manuscript.

## Conflict of Interest

The authors declare that the research was conducted in the absence of any commercial or financial relationships that could be construed as a potential conflict of interest.

## Footnotes

1 https://portal.gdc.cancer.gov/

2 http://cibersort.stanford.edu/

3 http://xCell.ucsf.edu/

4 http://gdac.broadinstitute.org/

5 https://cloud.genepattern.org/gp/pages/index.jsf

## Supplementary Material

FIGURE S1 The correlation between ATM and the mutation rates in the top 20 genes of the TCGA-Bladder cancer cohort. Blue indicates co-occurence; Brown indicates mutually exclusive. The color intensity indicates the correlation degree. ^∗^*P* < 0.05; ⋅*P* < 0.1.

FIGURE S2 Quantifying the infiltration of immune cells in the ATM-MT and ATM-WT tumors in the TCGA-BLCA cohort. **(A)** xCell analyses quantifying the proportion of immune cells in the ATM-MT and ATM-WT tumors in the TCGA-BLCA cohort. ^∗^*P* < 0.05; “ns”: *P* > 0.05. **(B)** MCP counter analyses quantifying the absolute abundance of immune and stromal cells in the ATM-MT and ATM-WT tumors in the TCGA-BLCA cohort. ^∗^*P* < 0.05; “ns”: *P* > 0.05.

FIGURE S3 Boxplot showing average changes in the expression levels of checkpoints genes between the ATM-MT and ATM-WT patients in the TCGA-BLCA cohort. ^∗^*P* < 0.05; “ns”: *P* > 0.05.

TABLE S1 Eight DNA damage repair (DDR) pathway gene sets. BER, base excision repair; DSB, double-strand break repair; FA, Fanconi anemia; HR, homologous recombination; MMR, mismatch repair; NER, nucleotide excision repair; NHEJ, non-homologous end-joining; SSB, single-stranded DNA binding.
