# Supplementary figures and images for: ATM Mutations Benefit Bladder Cancer Patients Treated With Immune Checkpoint Inhibitors by Acting on the Tumor Immune Microenvironment
Source: Front Genet. 2020 Aug 14;11:933. doi: 10.3389/fgene.2020.00933 (PMC7456912; doi:10.3389/fgene.2020.00933)

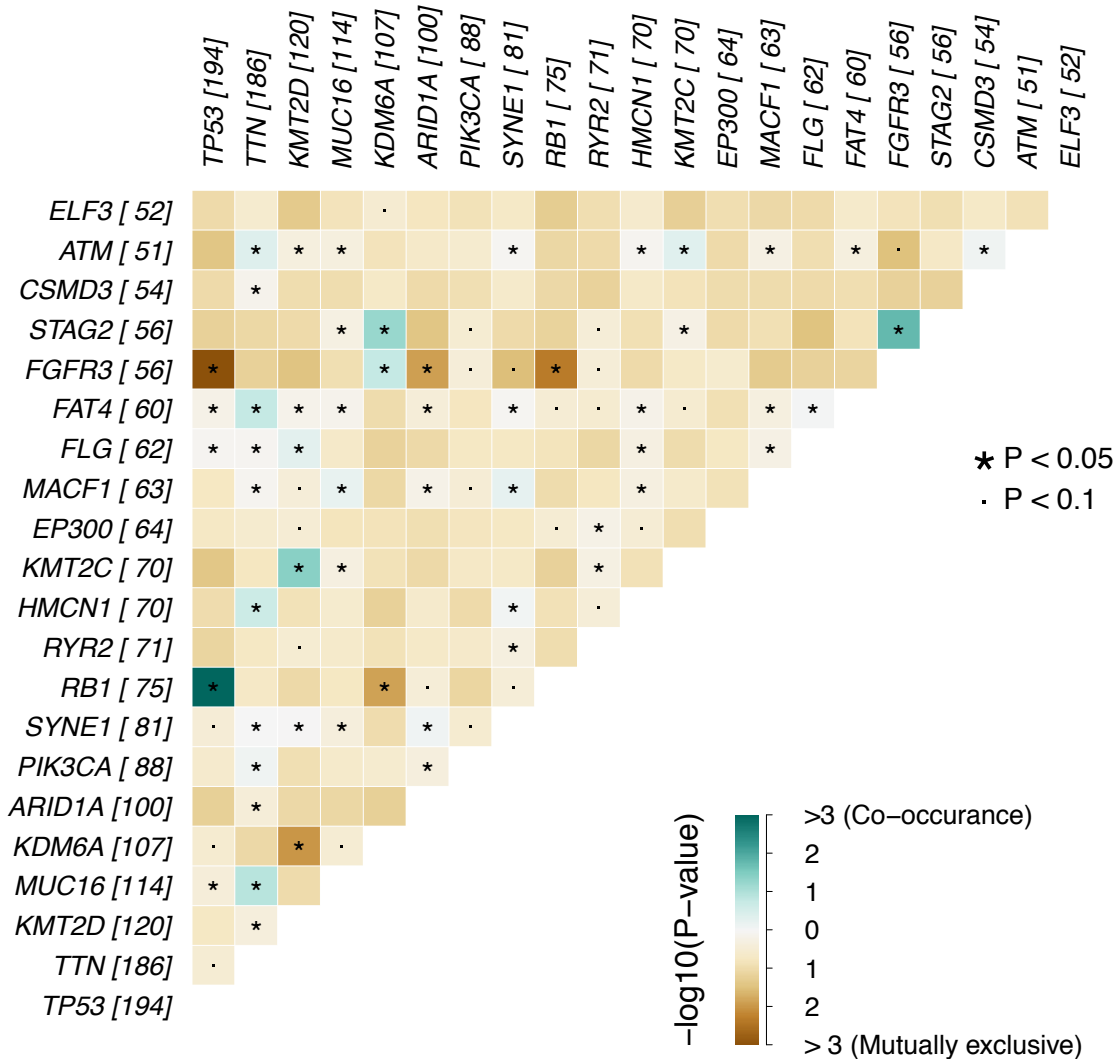

Supplement: FIGURE S1 — The correlation between ATM and the mutation rates in the top 20 genes of the TCGA-Bladder cancer cohort. Blue indicates co-occurence; Brown indicates mutually exclusive. The color intensity indicates the correlation degree. ∗P < 0.05; ⋅P < 0.1. [file Image_1.PDF]

A

ATM 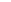 WT (355) 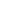 MT (52)

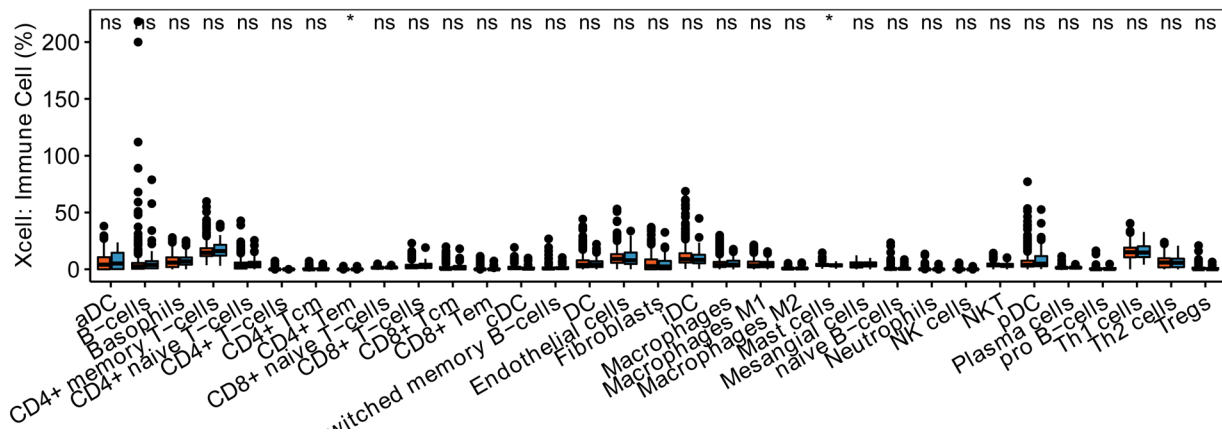

# B

ATM 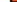 WT (354) 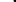 MT (51)

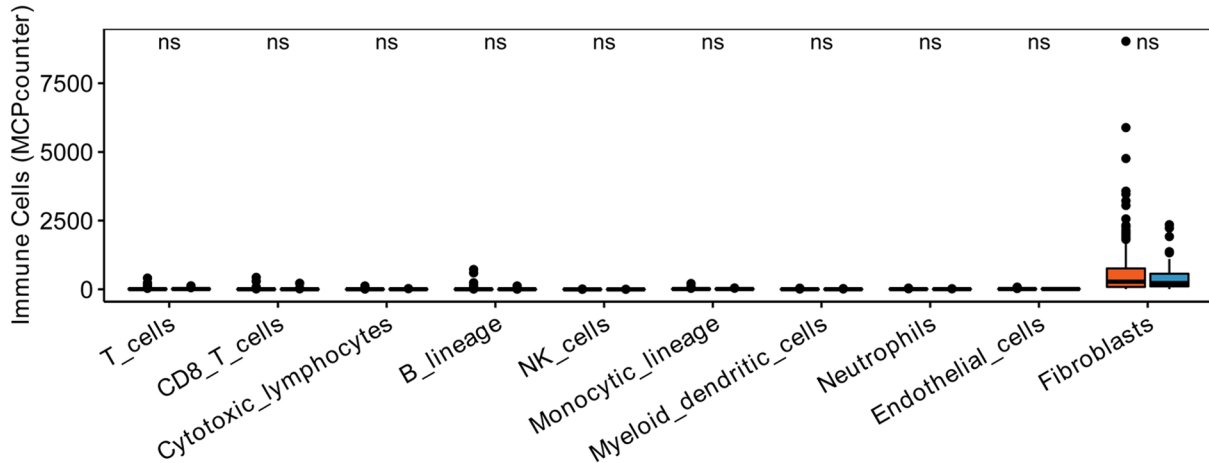

Supplement: FIGURE S2 — Quantifying the infiltration of immune cells in the ATM-MT and ATM-WT tumors in the TCGA-BLCA cohort. (A) xCell analyses quantifying the proportion of immune cells in the ATM-MT and ATM-WT tumors in the TCGA-BLCA cohort. ∗P < 0.05; “ns”: P > 0.05. (B) MCP counter analyses quantifying the absolute abundance of immune and stromal cells in the ATM-MT and ATM-WT tumors in the TCGA-BLCA cohort. ∗P < 0.05; “ns”: P > 0.05. [file Image_2.PDF]

ATM WT (354) MT (51)

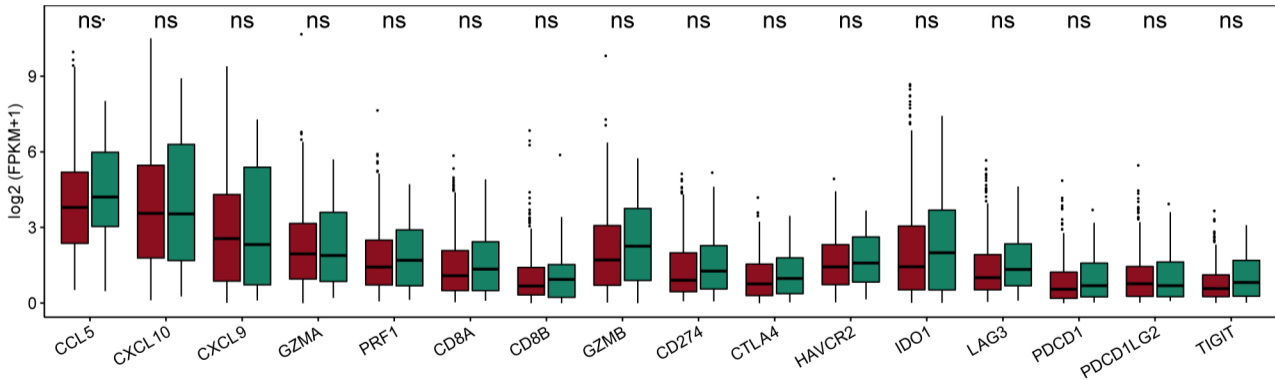

Supplement: FIGURE S3 — Boxplot showing average changes in the expression levels of checkpoints genes between the ATM-MT and ATM-WT patients in the TCGA-BLCA cohort. ∗P < 0.05; “ns”: P > 0.05. [file Image_3.PDF]
