# Supplementary material for: ATM Mutations Benefit Bladder Cancer Patients Treated With Immune Checkpoint Inhibitors by Acting on the Tumor Immune Microenvironment
Source: Front Genet. 2020 Aug 14;11:933. doi: 10.3389/fgene.2020.00933 (PMC7456912; doi:10.3389/fgene.2020.00933)
Supplement: Supplementary file 5 [file Table_2.docx]

Figure legends

Supplementary Figure 1. The correlation between ATM and the mutation rates in the top 20 genes of the TCGA-Bladder cancer cohort. Blue indicates co-occurance; Brown indicates mutually exclusive. The color intensity indicates the correlation degree. * p <0.05; · p <0.1.

Supplementary Figure 2. Quantifying the infiltration of immune cells in the ATM-MT and ATM-WT tumors in the TCGA-BLCA cohort.

1. xCell analyses quantifying the proportion of immune cells in the ATM-MT and ATM-WT tumors in the TCGA-BLCA cohort. * p <0.05; “ns”: p >0.05.
2. MCP counter analyses quantifying the absolute abundance of immune and stromal cells in the ATM-MT and ATM-WT tumors in the TCGA-BLCA cohort. * p <0.05; “ns”: p >0.05.

Supplementary Figure 3. Boxplot showing average changes in the expression levels of checkpoints genes between the ATM-MT and ATM-WT patients in the TCGA-BLCA cohort. * p <0.05; “ns”: p >0.05.

Supplementary Table 1. Eight DNA damage repair (DDR) pathway gene sets. BER, base excision repair; DSB, double-strand break repair; FA, Fanconi anemia; HR, homologous recombination; MMR, mismatch repair; NER, nucleotide excision repair; NHEJ, nonhomologous end-joining; SSB, single-stranded DNA binding.
